# Stakeholders’ perspectives of dental imaging in the current diagnostic radiography curriculum

**DOI:** 10.4102/hsag.v30i0.3149

**Published:** 2025-10-31

**Authors:** Keshini Govindasami, Shenuka Singh

**Affiliations:** 1 Discipline of Public Health, School of Nursing and Public Health, University of KwaZulu-Natal, Durban, South Africa; 2 Discipline of Dentistry, School of Health Sciences, University of KwaZulu-Natal, Durban, South Africa

**Keywords:** dental radiography training, scope of practice, stakeholder engagement and collaboration, document review, undergraduate curriculum

## Abstract

**Background:**

Dental imaging supports accurate diagnosis of orofacial anomalies and is included in diagnostic radiography training in South Africa (SA). Yet, there is limited published evidence on these professionals’ competencies to perform such tasks. It is unclear whether dental imaging is explicitly expressed in the undergraduate curricula for diagnostic radiography.

**Aim:**

To explore stakeholder perspectives (academics and Radiography and Clinical Technology [RCT] board members) on the provision of dental imaging within the current scope of practice and training for diagnostic radiographers, and to conduct a document analysis.

**Setting:**

In three selected provinces in SA: KwaZulu-Natal, Gauteng and Western Cape.

**Methods:**

An in-depth descriptive case study using exploratory, interpretivist design was conducted with purposively selected academics (*n* = 8) and RCT board members (*n* = 4) for diagnostic radiography, using semi-structured interviews. A curriculum review was conducted on publicly available documents. Data were triangulated and analysed using thematic and content analysis.

**Results:**

Key themes included the perceived understanding of the dental imaging scope of practice, training and limited exposure to undergraduate dental imaging training affects skills development. The limited availability of publicly accessible training documents created an unclear picture of the extent to which dental imaging is incorporated into the undergraduate curriculum.

**Conclusion:**

Noted inconsistencies between participants’ perspectives and the findings from document analysis, highlighting the need for greater stakeholder engagement and collaboration to define how dental imaging should be taught.

**Contribution:**

This study underscores the critical need for stakeholder collaboration in aligning dental imaging training and practice for diagnostic radiography.

## Introduction

Diagnostic radiographers worldwide are legally bound by a scope of practice that sets the regulatory framework under which they can practice, given their level of competence and educational training (Van De Venter & Friedrich-Nel 2021). Research (Crowling 2012; McNulty, England & Shanahan 2021) has shown that radiographers’ scope of practice and training vary significantly across countries because of clinical placement times, inclusion of skills labs and clinical supervision. The scope and training pertaining to diagnostic radiography in South Africa (SA) is vast and includes several areas of work in various healthcare settings (Health Professions Council of South Africa [HPCSA] 2020). Currently, in SA, diagnostic radiography practice is governed by a regulatory board known as the Board of Radiography and Clinical Technology (RCT) and falls under the HPCSA (2020).

Hudson et al. (2022) and Van De Venter and Engel-Hills (2022) have argued for the need for higher education programmes in SA to be more aligned with the needs of society and the workplace. Numerous studies have also shown that healthcare services fail because of the misalignment of health professionals’ education with the demands they encounter in practice (Crowling 2012; Sa Dos Reis et al. 2018). It is therefore imperative that the scope of practice is reviewed for its relevance and ability to meet the demands of the profession. At the same time, educational curricula should focus on developing clinically competent healthcare professionals who can navigate their professional scope across a wide range of healthcare settings (Hudson et al. 2022; McNulty et al. 2021).

Diagnostic radiographers are also required to work in dental imaging, an area vital to modern dentistry as it provides a platform for reliable and rapid diagnosis of several oral health conditions (Park & Yoon 2022; World Health Organization [WHO] 2022). One area that could contribute to an effective oral health system is revisiting the practice and training of diagnostic radiographers in dental imaging (Govindasami & Singh 2025). This proposal is supported by several studies that also highlight the need to develop innovative dental imaging education systems (Chen et al. 2022; Mahasneh et al. 2022). Dental imaging is within the scope of practice and training for SA’s diagnostic radiographers (HPCSA 2020); yet, there is limited published evidence on diagnostic radiographers’ knowledge and skills to perform such tasks. In addition, it is unclear if diagnostic radiographers are aware of the extent to which the scope of practice allows these tasks. Furthermore, there is no published evidence to the extent to which dental imaging is included in undergraduate curricula for diagnostic radiography.

### Objective

This study aimed to explore stakeholder perspectives (academics and RCT board members) on the provision for dental imaging in the current scope of practice and training for diagnostic radiographers, and to conduct a document analysis.

## Research methods and design

### Study design

This study used a descriptive case study approach, which is an effective method for conducting an in-depth analysis of a single unit such as an individual, a community or an organisation (Yin 2009). This design enabled the researcher to gain a comprehensive understanding of the subject, allowing for detailed description, interpretation and explanation of the research context or issue.

This was a qualitative study using an exploratory and interpretivist study design approach. The primary nature of qualitative research is exploratory, making it ideal for this study as it allowed the researcher to uncover meaning and understand stakeholders’ (academics and RCT board members) perceptions regarding diagnostic radiographers’ practice and training in dental radiography holistically (Baxter & Jack 2008:544–545).

In the first part of the study, two semi-structured interviews were conducted with stakeholders (academics and RCT board members) to explore their perspectives on dental radiography. Data from these interviews were interpreted for a deep and rich understanding of participants’ perceptions regarding dental radiography. The second part of the study focused on a document analysis of the undergraduate diagnostic radiography curriculum, conducted to identify whether there is a need to revisit the current training framework for dental radiography. This is presented as Part 1: Identified stakeholders’ perspectives on dental radiography and Part 2: Document Analysis Method.

### Part one: Identified stakeholders’ perspectives on dental imaging

#### Methods and study population

The study population consisted of academics training diagnostic radiographers and members of the Board of RCT, HPCSA. Two groups of participants were included: Group one represented academics from higher education institutions (HEIs) and Group two represented RCT board members. The first set of interviews was conducted with Group one (academics; *n* = 8), followed by interviews with Group two (RCT board members; *n* = 4).

#### Selection criteria for site selection

Academics working in the provinces of KwaZulu-Natal, Gauteng and the Western Cape were targeted, as these provinces have many health institutions offering oral healthcare diagnostic services (Bhayat & Chikte 2019). Therefore, diagnostic radiographers from these provinces were more likely to have exposure to dental imaging, providing greater insight to answer the study objectives. There is only one main HEI offering radiography training in KwaZulu-Natal and the Western Cape, and it was therefore included. In Gauteng, four HEIs offer radiography training, but only two were included, as they provide postgraduate training in specialised areas of diagnostic radiography (Computed Tomography [CT], Magnetic Resonance Imaging [MRI] and Mammography). Academics from these institutions were expected to provide deeper insight into dental radiography training within diagnostic radiography.

#### Study participant selection criteria, sampling and recruitment

Following gatekeeper permission from the HEIs and HPCSA and obtaining ethical clearance, purposive sampling was used for both groups. The researcher did not participate in recruiting participants. The *Protection of Personal Information* (POPI) *Act* was adhered to through the following recruitment strategies.

**Group one:** A sample of 20 academics was targeted in 2024. Selection criteria required that academics be involved in training diagnostic radiography students and have 1–10+ years of experience. Academics not involved in training were excluded. Study information and consent letters were emailed to academic managers for distribution. Eight academics consented to participate.

**Group two:** Radiography and Clinical Technology board members were contacted via HPCSA administrators, who provided potential participants’ contact information (Mndluli 2023). Selection criteria required diagnostic radiography training, ≥ 5 years’ experience and ≥ 3 years on the RCT board. In 2024, the board had 13 members, 5 were from the radiography discipline: 4 consented to participate.

Voluntary participation was ensured through signed informed consent. Interviews were conducted at pre-arranged times via Microsoft Teams, with individual links emailed to each participant.

#### Data collection

**FIGURE 1:**
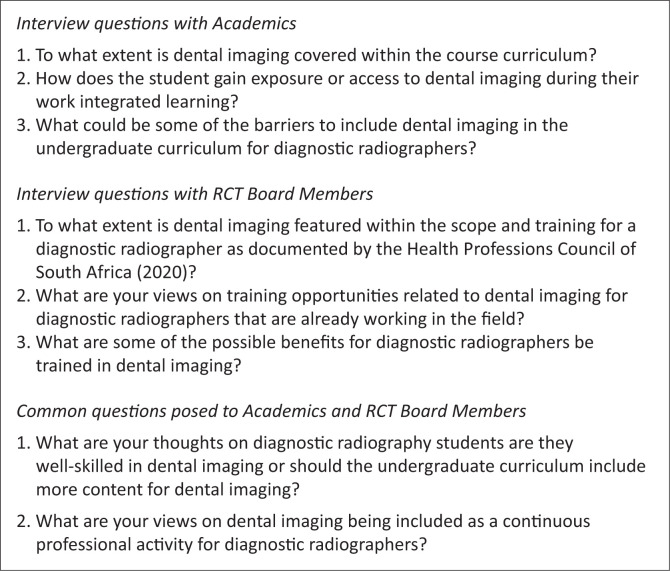
Interviews with academics and radiography and clinical technology board members.

**Group one:** A semi-structured interview schedule was used, with Section One covering demographics (sex: female, male, other; years of experience) and Section Two incorporating a Grand Tour Question: ‘*What are your perspectives regarding diagnostic radiographers’ training and practice in dental radiography?*’ Open-ended probing questions focused on student training and exposure (Figure 1).

**Group two:** A semi-structured interview schedule with Section One covering demographics (sex, years of experience, years on the RCT board) and Section Two using the same Grand Tour Question namely: What are your perspectives regarding diagnostic radiographers’ training and practice in dental radiography? Open-ended probing questions focused on scope of practice and training in dental imaging (Figure 1).

All interviews lasted 30 min – 45 min, were voice-recorded and transcribed verbatim. The data collection process ceased at data saturation. This was where information provided from the participants was becoming repetitive and redundant, and no new information that was relevant to the study purpose was emerging (Polit & Beck 2010).

#### Credibility of data collection – Part one

Credibility was established by using member checks, whereby the researcher emailed all participants their individual interview transcripts. Participants could review and confirm that the data captured during their interview were correct (Polit & Beck 2010). All interview transcripts were checked and analysed in conjunction with the supervisor and a data analyst experienced in coding, as a form of peer checking. Thereafter, an agreement was reached with respect to all emergent categories and themes. This was done to enhance the confirmability and validity of all emergent categories and themes (Polit & Beck 2010). Transferability was enabled through detailed description of data and further enhanced by comparing research findings with current published literature (Johnson & Onwuegbuzie 2016; Polit & Beck 2010). Confirmability was ensured by displaying direct quotations from participants (Johnson & Onwuegbuzie 2016; Polit & Beck 2010).

#### Analysis of interviews

All interview recordings were transcribed verbatim. The narratives obtained from Group One and Group Two were first read for familiarisation (Naeem et al. 2023). All narratives were read and re-read to identify key words or phrases, and to check for patterns. These key words or phrases were assigned to codes. Common codes were then grouped into categories, and common categories were formulated into themes (Johnson & Onwuegbuzie 2016; Naeem et al. 2023). All themes were reviewed for similarity and further grouped into overarching themes. Finally, compilation was confirmed through member checks, where interview transcripts were emailed back to participants to verify the data provided.

The original coding process was performed manually by the researcher with a data analyst experienced in coding, and all codes were independently checked by the supervisor and the data analyst. The researcher then drew conclusions and interpreted themes in relation to the study objectives (Bengtsson 2016; Naeem et al. 2023). This process constituted a thematic analysis.

#### Ethical considerations

Ethical approval to conduct this study was obtained from the Humanities and Social Sciences Research Ethics Committee University, University of KwaZulu-Natal (Reference no: HSSREC/00005832/2023). Confidentiality and anonymity of interviews were maintained by ensuring voluntary participation through signed informed consent. Pseudonyms were used on all interview transcripts to anonymise data. Data were stored on a secure, password-protected computer, accessible only to the researcher and supervisor (Johnson & Onwuegbuzie 2016; Polit & Beck 2010). Codes were assigned to the interviewees, for example, A1 for Academics and B1 for RCT Board members. University logos and identifying details were redacted, and documents were stored on a secure, password-protected computer.

### Part two: Document analysis method

A document review process was conducted by the researcher between April 2025 and June 2025. This was carried out to gain a richer understanding of the inconsistencies reported in Part One of the study findings (Andrade et al. 2018; Bowen 2009). In SA, diagnostic radiography training is currently offered at eight universities (Society of Radiographers 2021). The researcher conducted a document analysis on all publicly available documents, such as universities’ prospectuses, student study guides and undergraduate booklets sourced from these eight universities. Documents not older then 4years old were sourced (Andrade et al. 2018; Bowen 2009).

#### Selection criteria and data collection process

To identify the documents, an online search using the Google search engine was conducted. All available university curriculum documents (prospectuses, student study guides and undergraduate booklets) were sourced. Documents older than 4 years were excluded. The following search terms were used: ‘undergraduate diagnostic radiography curriculum offered in South Africa’ and ‘module and module contents offered for undergraduate diagnostic radiographers training’.

**FIGURE 2:**
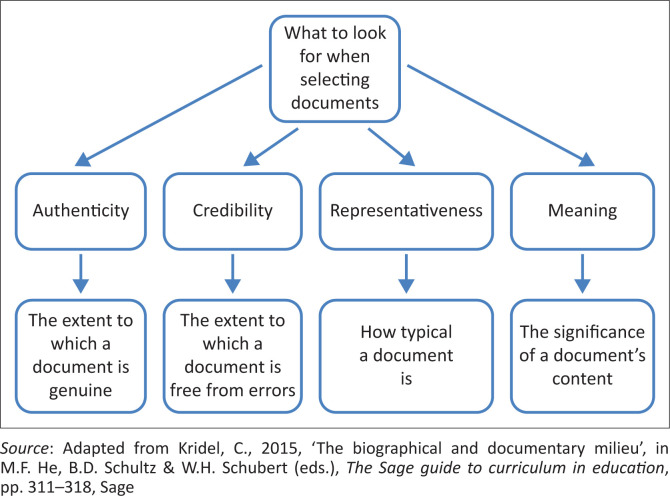
Factors for selecting documents.

All potentially sourced documents were evaluated based on the following criteria: authenticity, credibility, representativeness and meaning (Morgan 2022) (Figure 2). According to Flick (2018), using these selection criteria enhances the trustworthiness of sourced documents. The researcher ensured authenticity and credibility by sourcing only primary documents and checking that each document had the official university logo. Documents were also inspected for consistency in typography, formatting and style of language (Morgan 2022). Representativeness was ensured by sourcing official university documents not older than 4 years (2022–2025). The researcher further examined documents for literal meaning to understand the context in which the document was created (e.g., providing information for new prospective students or displaying module requirements for each study level) (Morgan 2022) (Figure 2).

#### Document review data analysis

The researcher used data extraction sheets adapted from previous studies (Andrade et al. 2018; Buchter et al. 2020). The first column, *File Name*, contained pseudonyms (A, B, C) to represent different universities. Additional columns, *Document Printed Year* and *Study Level*, reflected the authenticity and representativeness of documents.

Each curriculum document was read and re-read. Modules and their content were recorded under the columns *Module* or *Elective Module* and *Module Content* to enhance credibility. The researcher then evaluated whether each module contained content on imaging techniques or processes related to the oral cavity, teeth or dental imaging, as well as any key terms or phrases linked to dental imaging.

Data were grouped into two categories: Category 1: Imaging techniques and processes related to the oral cavity, teeth and dental imaging. Category 2: Key terms related to dental imaging. These were consolidated under the theme: Inconsistency in Module Offerings for Dental Imaging: The researcher then compared categories across modules at the same level (e.g., Level 2) and at different levels (e.g., Level 2 vs. Level 3). All categories were reviewed by the supervisor and a data analyst to strengthen credibility through peer review.

### Part one: Identified stakeholders’ perspectives on dental imaging

#### Results

The demographic profile of participants included academics, who were mainly female, with a diagnostic radiography qualification and experience training students in the diagnostic discipline. Their years of experience ranged from 3 to over 10 years. The RCT board members were also mainly female, with diagnostic radiography qualifications. Two members held dual qualifications, namely in diagnostic radiography and ultrasound. All members had over 20 years of experience and had served on the RCT board for approximately 4 to 5 years.

The study data uncovered the following main themes:

- Theme 1: Perceived understanding of the current dental imaging scope of practice and training.
- Theme 2: Limited exposure to dental imaging affects skills development.
- Theme 3: Increasing diagnostic radiographers’ clinical practice in dental imaging could have perceived benefits.
- Theme 4: Increased stakeholder engagement and collaboration for training alignment and skills development.

**Theme 1: Perceived understanding of the dental imaging scope of practice and training:** The majority of RCT board members agreed that dental imaging is included in the scope of practice for diagnostic radiographers. Both RCT board members and academics reported that, in industry, diagnostic radiographers are primarily limited to using pan-oral units and, in some practices, cone beam computed tomography (CBCT). Consequently, current qualified diagnostic radiographers and students are exposed to and trained on these modalities. Most academics and board members perceived that the current scope of practice and curriculum content adequately covers dental imaging in line with industry expectations for graduates, as articulated in the following statements:

‘In industry, radiographers and students are only exposed to pan oral work. There is no intra-oral dental equipment, and this affects how they gain clinical exposure.’ (A1)

‘Clinical practical exposure differs according to which hospital students may go to. Example: I was working at Hospital C. There was the pan oral X-ray machine in the X-ray Department, and the radiographers were performing pan oral imaging but not intra-oral. But at my current place of employment, Hospital B, the radiographers do not do the pan oral either, because we have a dental school within this hospital where all these dental X-rays are being performed.’ (B2)

‘I think currently the curriculum we cover is probably sufficient for a radiographer, because the dentist does their own periapical, bitewings, occlusal, and things.’ (A7)

‘So, in terms of radiographers’ X-raying of the individual teeth (intra-oral), to my recollection, the radiographers are not involved in that one, but mainly they do the pan oral.’ (B3)

However, there appeared to be some confusion, as not all RCT board members agreed on the full extent of the dental imaging scope for diagnostic radiographers. One RCT board member commented that if radiographers conducted intra-oral imaging without formalised training, they would be contravening the current scope of practice for diagnostic radiographers. Another board member observed that there is no independent licensing for dental radiographers in SA. This board member further reported that all members serving on the Board of RCT would need to revisit and clarify the scope of dental imaging practice in SA through further discussions, as articulated in the following quotes:

‘Dental imaging is part of the scope for the diagnostic radiographers but is limited to the use of panoramic machines, and they did not train to do intraoral dental work. Perhaps it might be informed by changes in the scope for dentists. Not sure? … I hope that the training radiographers receive from those dentists is a formalised one, which can be certified, to do intraoral radiography.’ (B1)

‘… And we need to get the blessing of that board to say that radiographers will be able to do intra-oral dental work and can therefore extend their work into this, right … that was previously done by the assistant dentist.’ (B2)

‘Challenge you probably maybe would find amongst maybe the dentist themselves who might view this as a scope creep.’ (B4)

Some RCT board members and academics felt that expanding the scope would ensure that the training curriculum is reviewed and aligned with international standards, as well as promote interprofessional collaboration and job satisfaction, as indicated in the following quote:

‘Patient centeredness with regards to radiographers and collaborating with dental professionals.’ (A8)

When academics and RCT board members were asked questions pertaining to dental image reporting as a form of role expansion, some stated that in SA, diagnostic radiographers are not allowed to perform formal image reporting. Instead, the concept of pattern recognition is introduced, where radiographers may comment on what they observe in radiographic images rather than provide a written report. According to the academics and RCT board members, this form of image pattern recognition is already included in the undergraduate general radiography curriculum, as illustrated in the following statement:

‘I think that’s a very small part of radiography in terms of dental imaging … more need for image reporting in general radiography pattern recognition. There is some pattern recognition already built into the undergraduate program; they do look at pattern recognition for dental imaging.’ (A7)

One RCT board member stated that Standard Operating Procedures (SOPs) are being developed by the HPCSA for image interpretation and recommended that dental image interpretation could be included in these SOPs.

**Theme 2: Limited exposure to dental imaging affects skills development:** Limited clinical training opportunities were considered one of the main barriers. Academics and RCT board members stated that most clinical sites could not accommodate students’ training needs. Limited resources, particularly dental imaging equipment in industry, restrict clinical exposure for undergraduate radiography students. Furthermore, academics reported that poor infrastructure for dental services in the public sector affects access to dental imaging, as most students are placed in public institutions for clinical work-integrated learning, as reflected in the following quotes:

‘I think infrastructure, which is currently crippling the dental practice and radiography in general, especially in the government hospitals where most of our students are situated …’ (A3)

‘… not all our clinical placement venues have a pan-oral machine, so that therefore limits the amount of practice that students gain or that they get.’ (A8)

‘… Practically, the students are not taught anything about dental imaging. The problem, or one of the main challenges that we are experiencing, is that we do not have dental equipment.’ (A6)

‘… Limited resources. I cannot speak for the other clinical instructor, but to my knowledge, we have no dental imaging equipment to train.’ (A7)

There appeared to be inconsistent exposure between students attending private sector institutions and those in the public sector. It was found that some students in the private sector had no exposure to dental imaging. An RCT board member also raised concerns about limited training opportunities, which could result in in-house clinical training for these professionals who may not be formalised or accredited:

‘… The problem with in-house training, if not formalised or accredited, is that it can cause radiographers to be found illegally doing intraoral dental work that is outside of their scope … ’ (B1)

Some participants stated that the full range of dental imaging was previously covered in the curriculum; however, in the current curriculum it is limited as alluded to by participants:

‘I do think that it’s been a little bit of a neglected area, and I think that it’s something I remember, as a student, doing quite a lot of dental imaging.’ (A6)

‘In my viewpoint, it does not cover all the areas that are currently available in the market for dental imaging.’ (B4)

**Theme 3: Increasing diagnostic radiographers’ clinical exposure in dental imaging could have perceived benefits:** Both academics and RCT board members stated that there would be several benefits if diagnostic radiographers had increased practical exposure to dental imaging. These benefits were perceived to include improved patient waiting times, enhanced access to dental care, and better radiation safety:

‘Improve patients access to services, acquire more knowledge and skills … allows the curriculum to be reviewed and updated with international trends.’ (A1)

‘Increased job satisfaction, improvement to patient care, minimize the treatment period and a reduced radiation dose to the patient that will be a benefit.’ (B4)

**Theme 4: Increased stakeholder engagement and collaboration for training alignment and skills development:** Both academics and RCT board members highlighted the need for more concerted stakeholder engagement, including dental professionals, to identify opportunities and align training needs. Participants stated that increased stakeholder engagement was necessary to address current infrastructure and equipment challenges, allowing students to gain adequate clinical access and exposure. This included the need for interprofessional collaboration with experts in dentistry to assist in teaching students and to support greater investment in Continuous Professional Development (CPD) activities directed towards dental radiography:

‘I think we must start working with dental professionals to place students, because, like I said, clinical competence and having hands-on experience in positioning is vital.’ (A6)

‘Recommendations are that we increase the knowledge base of radiographers … this could be done through the invitation of guest lecturers who specialise in this.’ (A1)

‘I do think that it would be a lovely opportunity to have some sort of continuous professional development, with dental professionals’ assistance, to forward dental radiography.’ (B2)

### Part 2: Document review analysis of results

The findings from the document review of publicly available curriculum documents revealed the following main theme: Inconsistency in Module Offerings for Dental Imaging. This theme comprised two main categories: Category 1: Imaging techniques and processes related to the oral cavity, teeth, and dental imaging. Category 2: Key terms related to dental imaging.

There was marked inconsistency between module offerings across different universities. Some universities’ module content reflected dental imaging processes and key terms associated with the oral cavity, teeth and dental imaging. For example, a comparison of modules from universities at similar study levels, showed that some institutions included dental imaging, whereas other universities did not mention it. In addition, no key terms related to dental imaging were included in some modules.

In comparing university modules where dental imaging processes were included, two observations were apparent. Firstly, there were differences in the study level at which dental imaging was offered. Secondly, one university covered dental imaging processes and key terms extensively, including dental imaging techniques, pattern recognition and biophysics with dental equipment.

For other universities, it was difficult to draw realistic conclusions because of limited publicly available module content. Reviewing the same institution’s modules between 2022 and 2025 showed that in 2022, dental imaging processes were not included, but by 2025, they had been incorporated.

It was also evident that some universities offered elective modules, such as Forensic Radiography or Mammography, for students interested in these fields. However, dental imaging or radiography did not feature as an elective.

## Discussion

The study’s findings revealed that, although dental imaging is included within the scope of practice and training for South African diagnostic radiographers, participants expressed that, in most radiology departments, clinical practice is limited to the use of extra-oral units such as pan-oral units and CBCT. Participants further cited that limited dental equipment, resources and access to these facilities affect how students gain work-integrated learning. Therefore, HEIs training diagnostic radiographers with access to dental hospitals are more likely to expose students to dental imaging practice. These findings are consistent with a study conducted in Nigerian training hospitals, which indicated that limited or absent dental equipment drastically affects students’ access to clinical training in dental imaging (Akpaniwo et al. 2018).

Kumsa et al. (2022) and Ganjitsuda (2025) stated that adequate clinical placements and exposure to specific radiographic modalities are essential for learning and acquiring key competencies in knowledge, skill and professional attributes required for clinical radiography. These authors suggested a need for a more concerted effort from all relevant stakeholders to limit potential barriers to aligning radiography training needs.

One RCT board participant believed that dental imaging is limitedly covered, excluding intra-oral dental imaging in the scope of practice for diagnostic radiography and, therefore, conducting it could be seen as illegal practice. However, the International Commission on Radiological Protection (ICRP): Medicine and Dentistry states that diagnostic radiographers are licensed operators of dental equipment (Smith 1989). The ICRP also indicates that training in dental imaging should align with national and local standards. In SA, the relevant national and local bodies such as the South African Health Products Regulatory Authority and the HPCSA, stipulate that the diagnostic radiographer’s scope of practice includes the full range of dental imaging techniques, including intra-oral imaging (HPCSA 2020; South African Health Products Regulatory Authority 2022). This indicates that all diagnostic radiographers are legally allowed to perform intra-oral dental imaging.

The findings regarding limited practice of intra-oral dental imaging are consistent with research from Jordan, where radiologic technologists (diagnostic radiographers) demonstrated limited knowledge of quality assurance tests for intra-oral dental imaging equipment, attributed to their limited exposure to such equipment (Govindasami & Singh 2025; Mahasneh et al. 2022). Similarly, studies conducted in Taiwan (Chen et al. 2022) reported that undergraduate training features limited dental imaging content and that there is no independent licensing system for dental radiographers. Other authors (Chen et al. 2022; Furmaniak, Kołodziejska & Szopiński 2016; Mahasneh et al. 2022) suggested that greater emphasis is needed to develop innovative dental imaging courses for diagnostic radiography students. Overall, these studies indicate that gaps in dental imaging practice and training are not unique to SA. Even internationally, there appears to be an unclear understanding of how dental imaging content and training are delivered. This highlights the need to review opportunities to better develop and align dental imaging training within diagnostic radiography programmes in SA (Govindasami & Singh 2025).

Some participants in this study perceived that undergraduate training sufficiently covers dental imaging for industry expectations, notably with the exclusion of intra-oral dental imaging. However, other RCT board members and academics promoted the need for continuing professional education, suggesting that graduates may not be fully equipped to meet the demands of professional practice. These findings contrast with studies by performed by McNulty et al. (2021) and Hudson et al. (2022), which emphasised that healthcare educational curricula should ensure students acquire the skills necessary to meet professional demands.

As observed in the document analysis results, three university modules included dental imaging processes, but the level of study varied. Some offered it in the second year, others in the third. This inconsistency may reflect differences in depth of coverage, as higher-level modules are expected to provide more extensive knowledge. One university extensively covered dental imaging techniques, pattern recognition, and biophysics with dental equipment. Although other universities may include dental imaging content within core modules such as *Radiographic Practice, Radiation Sciences*, or *Clinical Radiographic Practice*, dental imaging is not featured as a standalone module, and the level of study varies. In addition, dental imaging is not offered as an elective, unlike other options such as Forensic Radiography or Mammography. The limited clarity from publicly available curricula makes it difficult to determine the extent to which dental imaging techniques and processes are taught. Therefore, it is critical that all aspects of the required scope of practice are clearly articulated in undergraduate curricula to ensure transparency and identify gaps that institutions can address.

Some RCT board members and academics also highlighted potential scope creep, where intra-oral dental imaging was perceived as within the dental professionals’ domain. This, sometimes referred to as task shifting, occurs when professionals perceive that others are encroaching on their field of practice (Van De Venter & Engel-Hills 2022). However, this study did not include the views of dental professionals. Further research could explore these nuances.

This study suggested that increasing diagnostic radiographers’ clinical practice in dental imaging could have several perceived benefits, including improved patient waiting times, access to dental care, and increased radiation safety. These findings are consistent with both local and international studies emphasising that expanding the scope of practice can improve patient management and care (Van De Venter & Friedrich-Nel 2021; Hardy et al. 2016).

Radiography and Clinical Technology board members and academics supported adequate stakeholder engagement, including relevant groups such as dental professionals, to address industry needs and gaps in dental imaging practice and training. Dental imaging spans multiple disciplines such as dentistry, allied dentistry and radiology necessitating engagement from all relevant parties. Hendricks, Hartman and Olckers (2022) emphasised the importance of interprofessional teamwork and collaborative efforts. Such collaborative pedagogies should be considered for aligning the practice and training of dental imaging across disciplines, as transparent communication reduces potential errors (Hendricks et al. 2022; Kubik-Huch et al. 2010; Spencer 2022).

### Study’s limitations and strengths

This study was limited to diagnostic radiography professionals and did not include dental professionals. However, the findings provide insight into aligning dental imaging practice and training and may guide further research across professional groups.

It is acknowledged that while academics and RCT board members provided input on dental imaging, their perspectives were not necessarily the official positions of their institutions or the RCT board. The study aimed to capture participants’ understanding of dental imaging training at the institutional and regulatory level, but individual perspectives may not align with official positions.

## Conclusion

The study’s findings highlight inconsistencies between participants’ perspectives and the available document analysis. This indicates a need for greater stakeholder engagement and collaboration, particularly between regulatory bodies and training institutions, to ensure clear and explicit guidance on how dental imaging should be taught within training programmes.

### Recommendation for future studies

There is an urgent need for broader stakeholder engagement at a national level to address reported challenges and barriers to dental imaging training in diagnostic radiography.
